# Dehumanization of Older Family Members: Novel Determinants of Elder Abuse Proclivity by Caregivers

**DOI:** 10.1093/geroni/igab046.1250

**Published:** 2021-12-17

**Authors:** E-Shien Chang, Joan Monin, Daniel Zelterman, Naomi Isenberg, Becca Levy

**Affiliations:** 1 Weill Cornell Medicine, New York City, New York, United States; 2 Yale School of Public Health, New Haven, Connecticut, United States; 3 University of Wisconsin, Madison, Madison, Wisconsin, United States; 4 Yale University, Woodbridge, Connecticut, United States

## Abstract

Elder abuse affects one in six older persons globally. Three limitations converge to impede progress in prevention: most research is victim- rather than perpetrator-based; the reliance on explicit, self-reported factors; and failure to account for psychological factors that motivate abuse in the first place. The current study will be the first to address these gaps by examining whether family caregivers’ dehumanization of older persons, or the denial of humanness to older persons as one of the most hateful age stereotypes, could explain elder abuse proclivity. Implicit dehumanization of older persons was measured by a novel implicit-association-test developed for this study. Explicit dehumanization was measured by a semantic differential question widely used in the literature. We used the reliable and validated 8-item Caregiver Abuse Screen to measure elder abuse proclivity. In the final survey of 585 caregivers, dehumanization was found to be prevalent with 51% of the caregivers implicitly and 31% explicitly dehumanizing older persons. As predicted, implicit and explicit dehumanization uniquely contributed to elder abuse proclivity (OR=1.23, 95% CI=1.02-1.50, p=.03) and (OR=1.26, 95% CI=1.05-1.51, p=.01), respectively, after adjusting for relevant covariates including caregiver burden, and caregivers’ and care-recipients' health. Also as predicted, implicit dehumanization improved the prediction of abuse proclivity above and beyond the explicit dehumanization of older persons and caregiver burden. Socio-etiological models of elder abuse perpetration and corresponding prevention design should consider the inclusion of dehumanization as a key risk factor for abuse proclivity in family caregivers.

